# Body size from birth to middle age and the risk of hip and knee replacement

**DOI:** 10.1186/s12891-016-1105-9

**Published:** 2016-06-14

**Authors:** Bette Liu, Angela Balkwill, Jane Green, Valerie Beral, on behalf of the Million Women Study Collaborators

**Affiliations:** School of Public Health and Community Medicine, University of New South Wales, Samuels Building, Sydney, NSW 2052 Australia; Cancer Epidemiology Unit, University of Oxford, Oxford, OX37LF UK

**Keywords:** Hip replacement, Knee replacement, Osteoarthritis, Body mass index, Overweight, Obesity, Childhood

## Abstract

**Background:**

Information regarding the effects of body size in childhood and early adulthood on the risk of hip and knee replacement in later life is inconsistent. We aimed to assess their effect, taking into account body mass index (BMI) in middle-age.

**Methods:**

Prospective cohort (Million Women Study) of 791,034 women with information on birth weight, body size at age 10 and age 20, and current BMI (at mean age 59.5 years) were followed for 6.82 million person-years. Adjusted relative risks (RRs) and absolute risks of hospitalisations for hip or knee replacement surgery for osteoarthritis were estimated.

**Results:**

After a mean of 8.6 years follow-up, 17,402 women had a hip replacement and 18,297 a knee replacement. Between the ages of 50 and 79 years, absolute risks for women with current BMIs of <22.5 kg/m^2^ and 35 + kg/m^2^ were respectively 5.6 and 13.2 % for hip replacement; and 2.6 and 35.1 % for knee replacement. Within each category of current BMI, increasing body size at age 10 and at age 20 had comparatively small effects; there were no significant associations with birth weight. We estimate that 40 % of UK women with a BMI 35 + kg/m^2^ have either a hip or knee replacement between the ages of 50–79 years; this compares to just 10 % of UK women with a healthy BMI (<25 kg/m^2^).

**Conclusions:**

The effects of body size in childhood and early adulthood on the absolute risks of either a hip or knee replacement are minimal compared to the effect of adiposity in middle age.

## Background

In middle-aged adults, overweight and obesity are well recognised risk factors for hip and particularly knee joint osteoarthritis [1–4]. There is however little information on the effect of overweight and obesity in childhood and early adulthood on later life risk of osteoarthritis of these joints. Some have suggested that excess weight in childhood affects the risk of knee pain [5] and osteoarthritis in later life [6], others suggest that excess weight as a young adult plays an important role in the risk of hip and knee osteoarthritis and joint replacement [7–9], and others suggest that excess weight throughout life is most relevant [6, 10]. Childhood weight can be correlated with weight in both early adulthood [11] and in middle age [12] which makes it difficult to separate out the relative contribution of body size at different ages on the risk of hip and knee replacement for osteoarthritis. In this regard, studies with large numbers of events can help because rather than simply adjusting for highly correlated measures of body size at different ages, individuals can be cross-classified by their body sizes at different ages and risks compared between these cross-classified groups.

Distinguishing the relative contributions of body size during childhood and adulthood on later life risk of hip and knee joint replacement from osteoarthritis is important as this can inform how effective interventions to reduce weight in childhood, or as an adult, may be in preventing these highly prevalent and costly health conditions in the ageing population. This report aims to assess what contributions body size at different periods in early life and adulthood have on the subsequent risk of hip and knee joint replacements for osteoarthritis.

## Methods

The Million Women Study is a prospective study that recruited 1.3 million women aged between 50–69 from breast-screening clinics in England and Scotland from 1996 to 2001 [13]. Study participants completed a questionnaire providing information on socio-demographic characteristics, health behaviours and other medical history at recruitment. On average every three years since recruitment participants have been invited to complete an additional questionnaire. These questionnaires are available at www.millionwomenstudy.org. Between 1999 and 2004, 866,000 women answered a resurvey questionnaire (baseline for this report) which asked for the first time about the women’s birth weight and their body size when they were young. Specifically women were asked: ‘How much did you weigh when you were born?’; ‘When you were about 10 years old, compared with average, would you describe yourself as: thinner? plumper? about average?’; and ‘What size clothes did you wear when you were about 20 years old?’ with options to respond: 8 or less, 10, 12, 14, 16, or 18+. We have previously reported on the accuracy of the responses by comparing them with birth weight recorded at the time of their birth, and height and weight measured at ages 11 and 20 years in a subset of 541 study participants who were also participants in the National Survey of Health and Development (NSHD) birth cohort [14]. Self-reported data on body size in earlier life in the Million Women Study were well correlated with measured values at the equivalent ages (correlation co-efficients 0.78, 0.51 and 0.63 for birth weight, size at 10 and size at 20 respectively). Current BMI calculated from self-reported data from the Million Women Study participants is also highly correlated with that calculated from measured height and weight (Pearson’s correlation 0.85) [15].

As described previously, Million Women Study participants have been followed up for deaths and cancers by flagging their records on the respective registries [13]. They have also been linked to national hospital admission databases (the Hospital Episode Statistics for England and the Scottish Morbidity Records for Scotland) using the participant NHS number, a unique personal identifier, as well as their date of birth and other identifying details [16]. These databases contain a record of every NHS-funded inpatient hospital admission from April 1997 in England and January 1981 in Scotland. For each hospital record up to 20 diagnoses and up to 24 procedures are coded according to the International Classification of Diseases version 10 (ICD-10) and the Office of Population Censuses Classification of Surgical Operations and Procedures version 4 (OPCS-4), respectively. Data for this report were available up until 31 December 2008 in Scotland and 31 March 2011 in England.

We identified participants as having a hip replacement for osteoarthritis if they had a hospital record with an OPCS-4 code for a primary total hip replacement (W37.1, W38.1, W39.1) and the corresponding main diagnosis field indicated hip osteoarthritis (ICD-10 M16). Participants were considered to have had a knee replacement for osteoarthritis if they had a hospital record with an OPCS-4 code for a primary total knee replacement (W40.1, W41.1, W42.1) and the corresponding main diagnosis field indicated knee osteoarthritis (ICD-10 M17).

### Statistical analysis

Women were excluded if prior to baseline they had an OPCS-4 code for any hip or knee replacement (OPCS-4 codes W37–W39 and W40–W42), or if they had reported a previous hip or knee replacement. Person-years were calculated from baseline to the date of first hospital admission for the joint replacement, date of death or the last date for which the hospital records were complete, whichever came first.

We classified women into categories of baseline BMI (calculated by dividing their self-reported weight in kilograms reported at return of the baseline questionnaire by their height in meters squared reported at recruitment to the study), and also cross-classified baseline BMI (in three categories: <25, 25- < 30, 30+ kg/m^2^) with each division of reported size when they were younger. For birth weight this was <2.5, 2.5- < 3.5 and 3.5+ kg; for size at age 10 this was thin, average and plump; for clothes size at age 20 this was small (less than size 12), medium (size 12) and large (sizes 14+). Cox proportional hazards models were used to estimate the relative risk of hip or knee replacement according to these categories cross-classified with baseline BMI. Analyses were initially adjusted for attained age, region of recruitment (10 regions), socioeconomic status (in quintiles based on the Townsend index of deprivation [17]), and then in addition, smoking (never, past, current), alcohol consumption (<3, 3–7, 8+ units per week), height (<160, 160- < 165, 165+ cm), use of hormone replacement therapy (never, past, current), parity (none, 1, 2+), age of menarche (<12, 12, 13, 14+ years), strenuous exercise (<=1, > 1 times weekly). Region of recruitment, socioeconomic status, height, parity, menarche and strenuous exercise were based on that reported at recruitment while smoking, alcohol consumption and hormone replacement, therapy use were based on the second questionnaire (baseline). For adjustment variables, missing data were included as a separate category in analyses.

We also conducted sensitivity analyses where we defined the outcomes as hospitalisation for a primary total hip or primary total knee replacement regardless of whether the accompanying primary diagnosis was osteoarthritis. Absolute risks of hip and knee replacement were also estimated according to BMI at baseline and sizes when young, adjusting for attained age and the other characteristics described above. Where more than two groups of body size were compared, variances were estimated by treating the relative risks as floating absolute risks [18]. This method does not alter the relative risks, but enables valid comparisons between any two exposure groups, even when neither group is the reference group. All analyses were conducted using STATA version 13.1.

## Results

Following exclusions, there were 791,034 women in the analyses. The average age at baseline was 59.5 years (SD 4.9) at which time 38 % were overweight (BMI 25- < 30 kg/m^2^) and 17 % were obese (BMI 30 + kg/m^2^). Table 1 shows the characteristics of women overall and by their reported body size at different ages (birth weight, size at 10 and 20 years, BMI at baseline (around age 60)). The characteristic most strongly related to birth weight was adult height. Women who reported being plump at age 10 or comparatively large clothes size at age 20 had a higher average BMI at baseline. Baseline BMI varied with almost all the variables in Table 1.

**Table 1:** Characteristics^a^ of study participants according to body size at birth, childhood, early adulthood and middle age

	At birth (kg)			Relative size at age 10 years			Relative size at age 20 years			BMI (kg/m^2^) at about age 60 years			Total
	<2.5	2.5- < 3.5	3.5+	Thin	Average	Plump	Small	Medium	Large	<25	25- < 30	30+	
Age (years) at baseline, mean (SD)	59.4 (4.8)	59.0 (4.8)	59.2 (4.9)	59.7 (4.9)	59.6 (5.0)	58.7 (4.8)	59.1 (4.8)	59.6 (4.9)	59.8 (5.1)	59.4 (5.0)	59.7 (4.9)	59.4 (4.8)	59.5 (4.9)
BMI (kg/m^2^) at baseline, mean (SD)	26.4 (4.9)	26.0 (4.5)	26.4 (4.8)	25.5 (4.3)	25.9 (4.3)	28.0 (5.5)	24.5 (3.7)	25.6 (3.9)	28.3 (5.3)	22.5 (1.7)	27.1 (1.4)	33.9 (3.8)	26.1 (4.6)
Highest socioeconomic tertile, % (N)	31.7 (19,115)	35.0 (84,935)	34.2 (50,239)	32.5 (80,151)	34.4 (143,760)	32.2 (37,814)	34.6 (81,086)	34.4 (103,215)	31.3 (76,102)	36.5 (131,033)	32.8 (96,469)	26.7 (35,213)	33.5 (262,715)
Height (cm), mean (SD)	160.2 (6.7)	162.1 (6.5)	163.9 (6.6)	162.6 (6.9)	162.2 (6.5)	161.9 (6.7)	159.9 (6.2)	162.4 (6.3)	164.3 (6.8)	163.3 (6.5)	161.7 (6.4)	160.6 (7.0)	162.3 (6.7)
Current smoker, % (N)	12.4 (7,396)	11.3 (27,134)	12.1 (17,638)	10.6 (25,924)	12.3 (50,908)	14.8 (17,231)	12.9 (29,910)	11.8 (34,998)	11.9 (28,713)	13.8 (49,008)	11.3 (33,025)	9.5 (12,397)	12.2 (94,430)
Alcohol (drinks pw), mean (SD)	4.8 (10.3)	5.4 (9.6)	5.4 (9.7)	5.1 (10.0)	5.2 (9.7)	5.1 (9.9)	5.6 (10.1)	5.4 (10.0)	4.5 (9.4)	5.7 (10.0)	5.1 (9.9)	3.8 (9.1)	5.2 (9.9)
Strenuous exercise > once a week, % (*N*)	22.2 (13,051)	23.8 (56,974)	23.9 (34,562)	22.1 (53,342)	23.3 (95,787)	22.5 (25,977)	23.7 (54,594)	23.4 (68,989)	21.2 (50,630)	27.0 (95,066)	21.0 (60,546)	15.7 (20,217)	22.8 (175,829)
Birth weight (kg), mean (SD)	2.0 (0.4)	3.1 (0.3)	4.0 (0.4)	3.1 (0.7)	3.3 (0.7)	3.4 (0.7)	3.1 (0.7)	3.2 (0.7)	3.3 (0.7)	3.2 (0.7)	3.2 (0.7)	3.2 (0.8)	3.2 (0.7)
Menarche age <12 years, % (*N*)	24.0 (14,352)	21.1 (50,852)	21.5 (31,243)	24.0 (14,352)	21.1 (50,852)	21.5 (31,243)	17.3 (40,198)	19.2 (56,937)	25.0 (60,318)	15.9 (56,446)	21.8 (63,599)	29.8 (38,896)	20.4 (158,941)
Parity, mean (SD)	2.1 (1.2)	2.1 (1.2)	2.1 (1.2)	2.1 (1.2)	2.1 (1.2)	2.0 (1.2)	2.1 (1.2)	2.1 (1.2)	2.1 (1.2)	2.0 (1.2)	2.1 (1.2)	2.3 (1.3)	2.1 (1.2)
Ever user of HRT, % (*N*)	54.8 (32,621)	55.5 (133,676)	54.8 (79,749)	57.2 (139,309)	52.8 (218,500)	52.9 (61,613)	59.3 (137,799)	54.3 (161,254)	49.2 (118,236)	55.2 (196,036)	54.5 (158,706)	50.9 (66,406)	54.2 (421,148)
Women, (*N*)	60,728	244,507	147,792	248,149	420,954	118,428	236,233	302,100	245,081	361,418	296,682	132,934	791,034
Hip replacement													
Cases (*N*)	1,338	5,129	3,258	4,918	9,346	3,063	3,529	6,261	7,424	6,273	7,056	4,073	17,402
Followup (years), mean (SD)	8.6 (2.1)	8.6 (2.0)	8.6 (2.0)	8.6 (2.0)	8.6 (2.1)	8.6 (2.1)	8.6 (2.0)	8.6 (2.1)	8.6 (2.1)	8.7 (2.0)	8.6 (2.1)	8.5 (2.1)	8.6 (2.1)
Knee replacement													
Cases (*N*)	1,537	5,283	3,420	6,061	9,187	2,970	3,621	5,953	8,525	3,532	7,395	7,370	18,297
Followup (years), mean (SD)	8.6 (2.1)	8.6 (2.0)	8.6 (2.0)	8.6 (2.1)	8.6 (2.1)	8.6 (2.1)	8.6 (2.0)	8.7 (2.0)	8.6 (2.1)	8.7 (2.0)	8.6 (2.0)	8.4 (2.2)	8.6 (2.1)

^a^Characteristics were reported at baseline, that is, at the same time as body size was reported except for socioeconomic status, height, strenuous exercise, menarche and parity which were based on data reported at recruitment about 3 years earlier

**Fig. 1:**
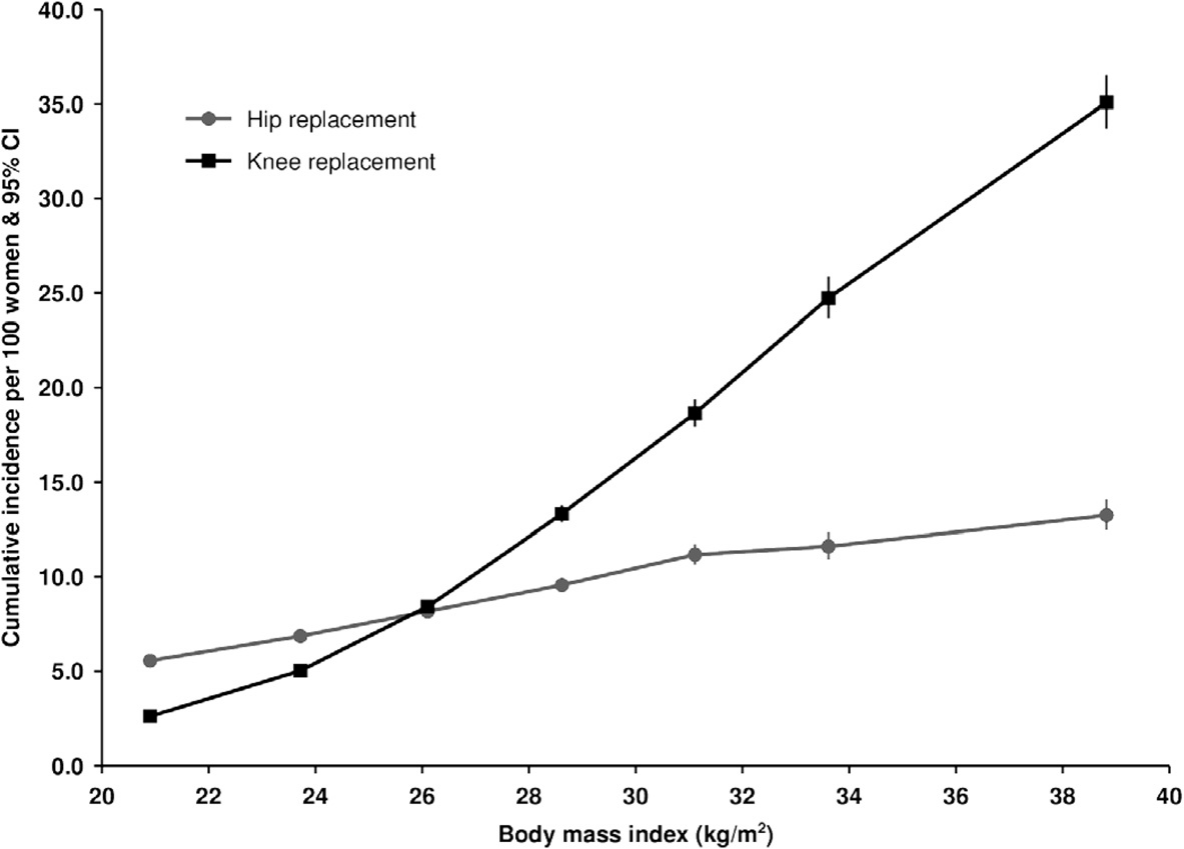
Cumulative incidence* of hip and knee replacement for osteoarthritis per 100 women at age 50–79 years by body mass index. Incidence plotted against mean BMI in each group. *Incidence rate adjusted for attained age, region of residence, socioeconomic status, smoking, alcohol use, height, hormone replacement therapy use, parity, age of menarche, strenuous exercise

Over 6.82 million person-years of follow-up (average 8.6 years per woman), 17,402 women had a first hip replacement and 18,297 had a first knee replacement. The risk of both hip and knee replacement among women increased with increasing BMI at baseline and the increase was much greater for knee than for hip replacements (Fig. 1). Women with a BMI of 26.1 kg/m^2^, the average BMI in the cohort at baseline, had similar cumulative risks from age 50 to age 79 both for hip replacement (8.2 %) and for knee replacement (8.4 %). However comparing women with BMIs of <22.5 kg/m^2^ and 35+ kg/m^2^ at baseline, the risks of hip replacement increased more than 2-fold, from 5.6 to 13.2 %, whereas for knee replacement the corresponding increase was 13-fold, from 2.6 to 35.1 %.

The relative risks (RRs) of joint replacement were estimated in minimally adjusted models (age, region of residence, socioeconomic status) and fully adjusted models (see methods for all characteristics included). There was little attenuation of the RRs between the two models and only the fully adjusted risks are shown and reported here.

**Fig. 2:**
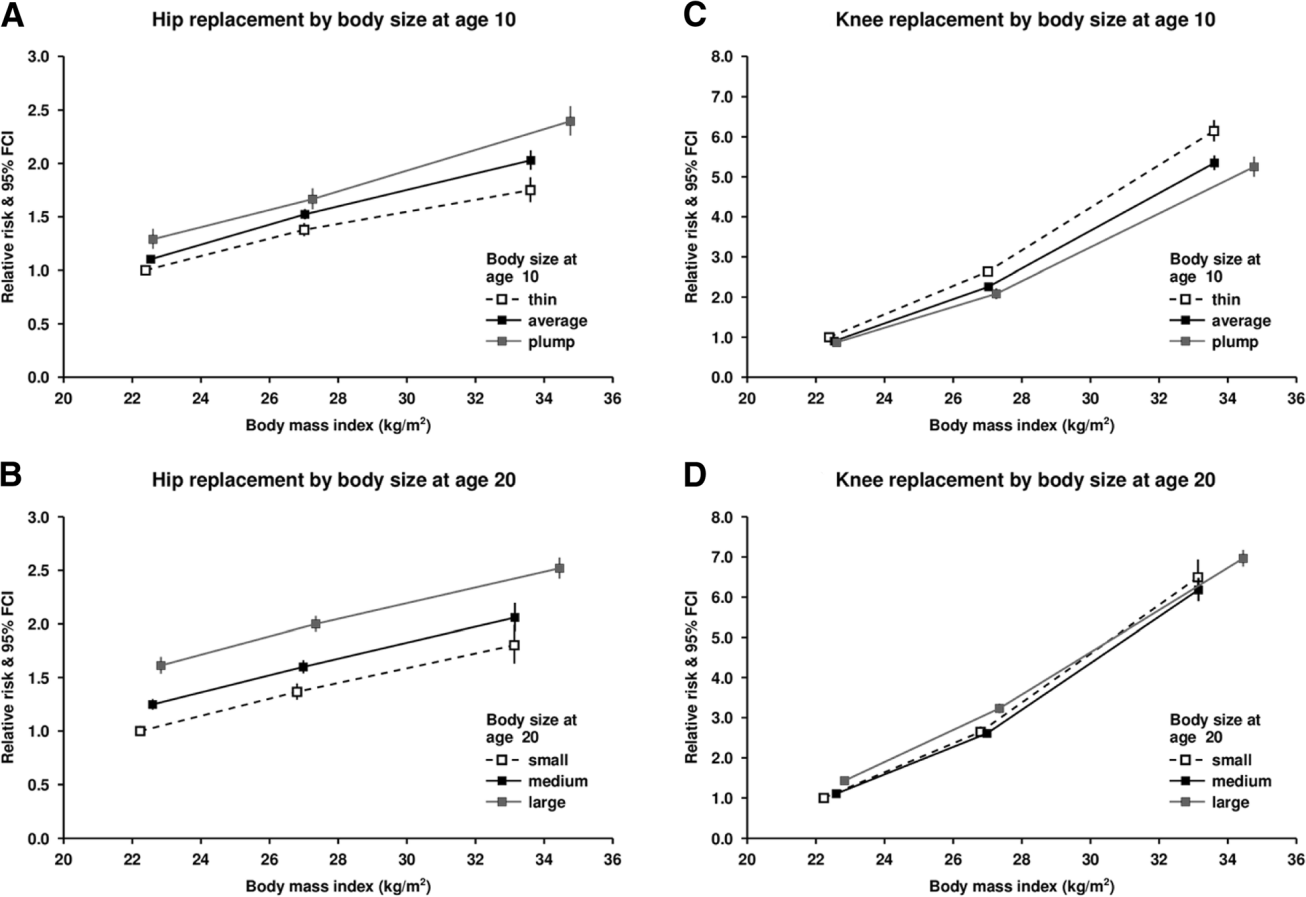
Relative risk of hip and knee replacement by body mass index at around age 60 years and reported body size at age 10 and 20 years. Relative risks plotted against mean BMI in each group

Figure 2 (and Appendix) shows RRs for hip and knee replacement by baseline BMI, cross-classified by body size at age 10 and 20. The pattern of risk by body size at different ages differed by the joint examined. The RRs of hip replacement increased with increasing size at age 10 and at age 20 years, as well as increasing with baseline BMI. Compared to women with a BMI <25 kg/m^2^ who reported being thin at age 10, the RR of a hip replacement in those with BMI 30+ kg/m^2^ who reported being thin at age 10 was 1.75 (1.64–1.87); for those with BMI 30+ kg/m^2^ who reported being plump at age 10 it was 2.40 (2.26–2.54); Fig. 2a. Increase in size at age 20 had almost as great an effect on the RR of hip replacement as increases in baseline BMI. For example, compared to women with a BMI <25 kg/m^2^ and small clothes size at age 20, the RR of women with a baseline BMI 30+ kg/m^2^ and small clothes size was similar to that in women with baseline BMI <25 kg/m^2^ and large clothes size at age 20 (RR 1.80 (1.63–1.99) and 1.61 (1.54–1.69) respectively); Fig. 2b.

For knee replacements any difference in RRs by body size at age 10 or at age 20 were substantially less than the effects of baseline BMI. For example compared to women with a BMI <25 kg/m^2^ and small clothes size at age 20, the RR of women with a baseline BMI 30+ kg/m^2^ and small clothes size was 6.49 (6.08–6.94), whilst for women with a baseline BMI <25 kg/m^2^ and large clothes size the RR was 1.43 (1.34–1.53); see Fig. 2d. In addition for reported size at age 10, if any effect was observed, it was in the opposite direction to that for hips (ie. in each category of baseline BMI, the RRs were smaller in those reporting being plump at age 10 years than in those reporting being thin; Fig. 2c).

**Fig. 3:**
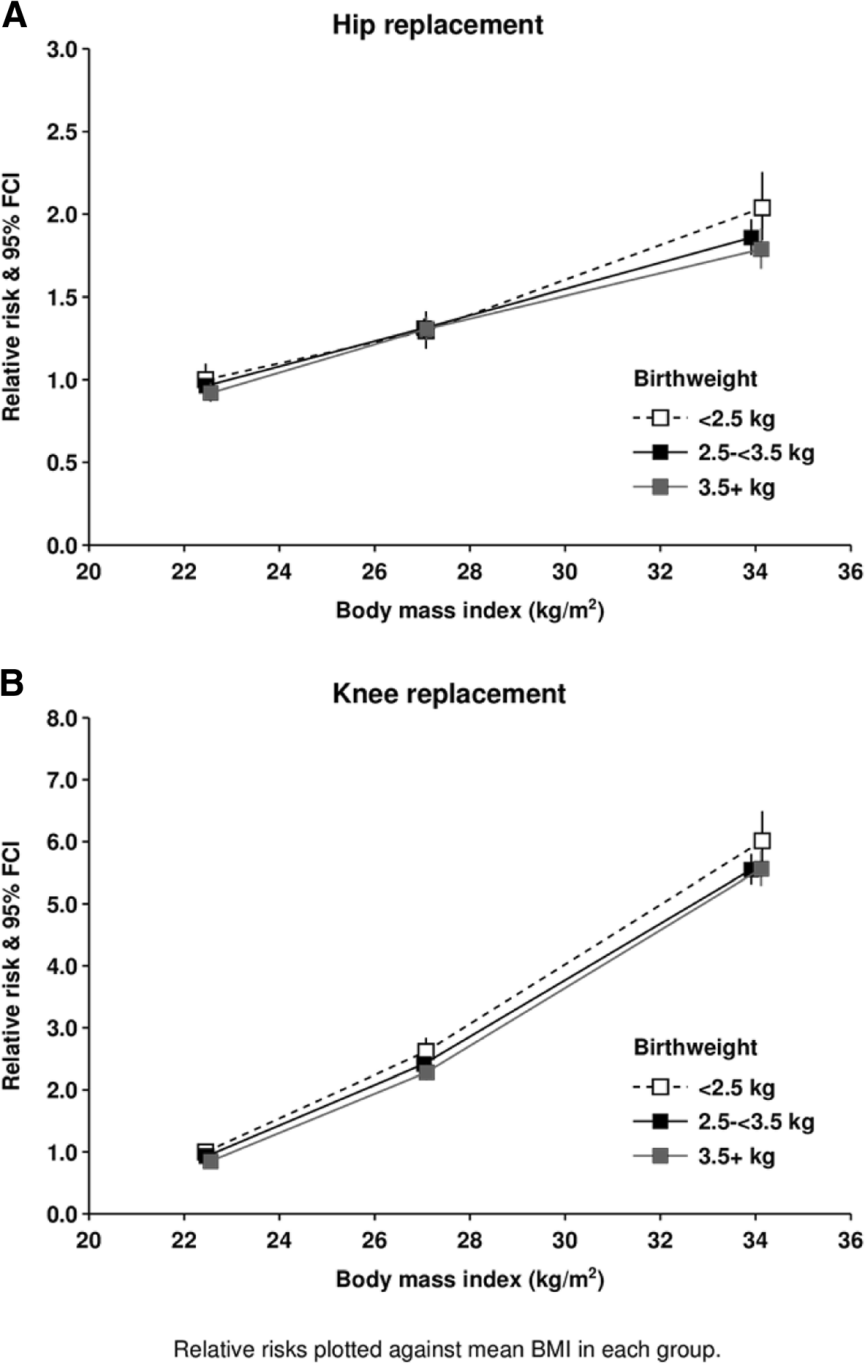
Relative risk of hip and knee replacement by body mass index at around age 60 years and reported birth weight. Relative risks plotted against mean BMI in each group

The RRs of hip and knee replacement by baseline BMI and categories of birth weight (<2.5, 2.5- < 3.5, 3.5+ kg) are shown in Fig. 3 and the Appendix. Within each category of baseline BMI, there was minimal variation of risk by birth weight.

In the sensitivity analyses, we included all primary total hip (*n* = 20,542) and total knee joint replacements (*n* = 20,357) in our outcome definition regardless of whether or not there was also a diagnosis code for osteoarthritis. The RR estimates in the cross-classified categories of baseline BMI and birth weight, and size at age 10 and age 20 were slightly attenuated but the patterns were similar to those in Figs. 2 and 3 (data not shown).

**Fig. 4:**
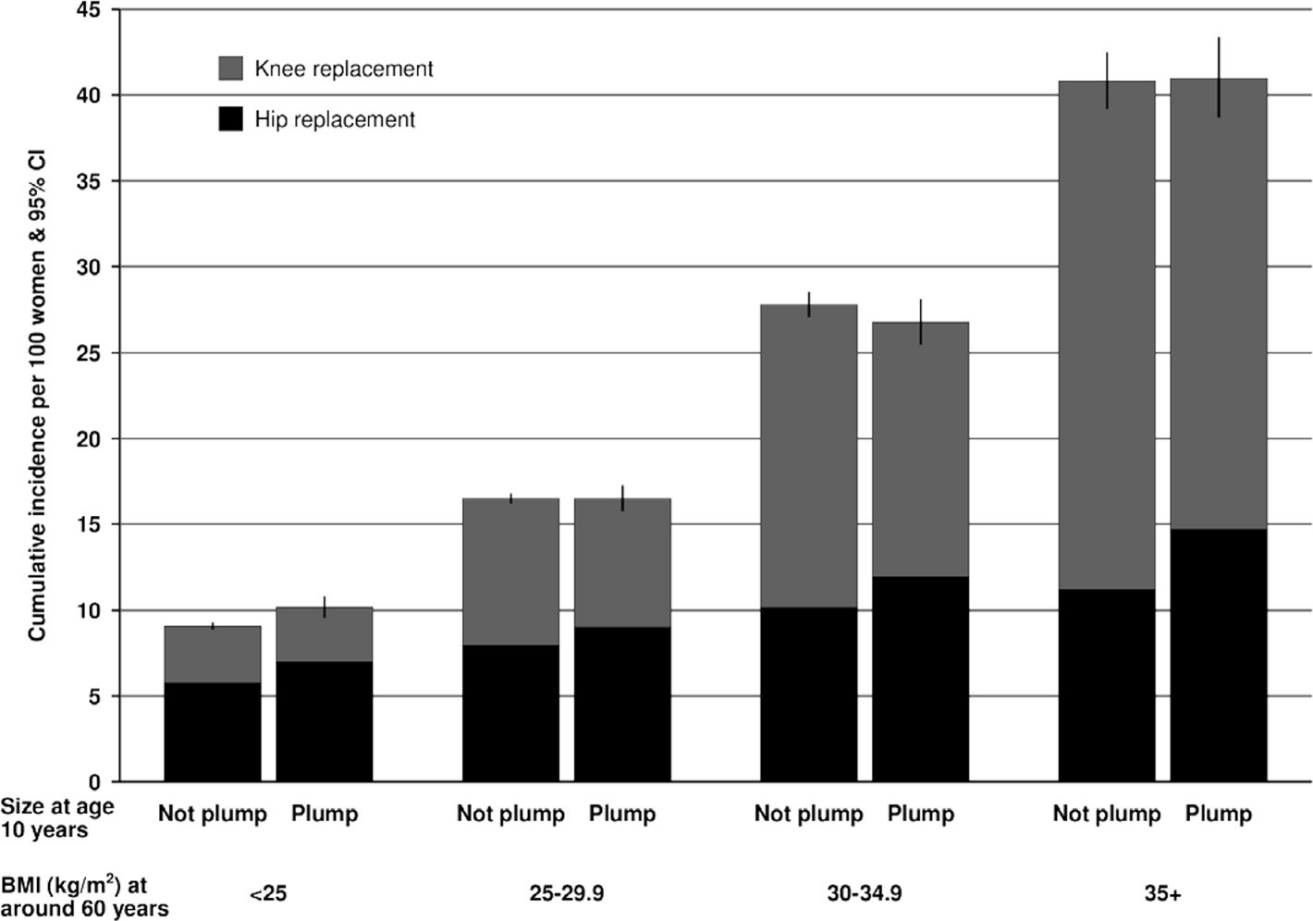
Cumulative incidence* of hip or knee replacement for osteoarthritis per 100 women at age 50–79 years by body mass index at around age 60 and reported size at age 10. *Incidence rate adjusted for attained age, region of residence, socioeconomic status, alcohol use, height, hormone replacement therapy use, parity, age of menarche, strenuous exercise

Figure 4 shows the absolute risk of hip or knee replacement for osteoarthritis from age 50 to 79 years by baseline BMI (BMI at around age 60 years) and size at age 10 years (plump or not plump). The absolute risk of having either of the large lower limb joints (hip or knee) replaced for osteoarthritis increased with baseline BMI, from 10 % of those with a BMI <25 kg/m^2^ to 40 % of those with a BMI of 35 kg/m^2^ or greater. A greater proportion of joint replacements in women with BMI <25 kg/m^2^ were for the hip, whilst knee replacements contributed a much greater proportion among the obese women. Among women with the same baseline BMI, there was little difference in the absolute risk between those who were plump or not plump at age 10.

## Discussion

In this large cohort of middle-aged women (average age at baseline almost 60 years) we found that, after allowing for the effect of their current BMI, birth weight and body size in childhood and early adulthood had relatively little effect on the risk of a knee replacement for osteoarthritis; for hip replacements body size in childhood and early adulthood somewhat increased the risks at every level of current BMI. Our results suggest that it should be possible to prevent a substantial number of hip and particularly knee joint replacements in overweight children and young adults if weight reduction can be achieved before reaching middle-age.

Previous reports are inconsistent regarding the effects of early life weight and/or body size on the risk of hip or knee osteoarthritis or replacement but not all of these studies accounted for adult BMI in their analyses [1, 9]. Some have concluded that BMI in early adulthood (around age 18 years) contributes more than recent BMI to the risk of hip replacement for osteoarthritis [7, 9] and that prevention strategies should be focussed on the young [1]. Others have concluded that after considering BMI in middle-age, early childhood and adolescent size has little bearing on the risk of later life knee osteoarthritis [6] or knee pain [5]. Studies examining both the hips and the knees have variously reported that larger size in early adulthood increases the risk of both hip and knee osteoarthritis [10] and of hip and knee replacement [8], and that larger early adulthood size increases the risk of knee but not hip osteoarthritis [19]. One of these studies also suggested that the effect of BMI on hip and knee replacement risk in middle-age was stronger than that in early adulthood and that the effects were greater for knees than for hips [8]. Many previous studies were retrospective in design, and could be biased by differential selection or recall, or had small sample sizes. Most did not estimate absolute risks of joint replacements in relation to body sizes at different ages, nor had they sufficient statistical power to robustly examine differences across both BMI in the middle-age and at earlier ages.

In our analyses, the effect of body size when young on the cumulative absolute risk of either a hip or knee replacement was far outweighed by the effect of BMI in middle-age (see Fig. 4). However, as reported in some other studies, there were some differences between hip and knee replacements. For hip replacements, larger body size at age 10 and particularly age 20 resulted in increases in relative risks at every level of BMI in middle-age. This pattern is consistent with a hypothesis that cumulative weight-bearing over time influences the risk of hip osteoarthritis. For knee replacements, earlier life weight had relatively little contribution to the risk of knee replacement compared with BMI at baseline. For a given baseline BMI, we did observe a small difference in risk by size at age 10 years (Fig. 2c), but this was in the opposite direction to that for the hips, with women who were plumper at age 10 having slightly lower risks of knee replacement that those who were thinner at age 10. These findings are counter to a hypothesis of cumulative weight-bearing contributing to risk of knee osteoarthritis and replacement and suggest that the mechanisms that lead to osteoarthritis of the hip and knee differ.

Our study strengths include the prospective design, large sample size, and long and virtually complete follow-up for study participants. In addition, the linkage to administrative hospital records for joint replacements was done independent of the study investigators. The self-reported earlier life size and BMI used in this report have been previously validated against physical measures collected at the equivalent ages, and have shown to compare well [14, 15]. In addition, as these data were collected prior to the outcomes of interest, joint replacement, we would not expect differential reporting by outcome. We were able to adjust our analyses for a range of potential confounders such as age, socioeconomic status, region, smoking, physical activity but found that these had minimal effect. We did not have information on joint injury in earlier life although other studies suggest this would not affect a large proportion of the cohort [19]. We were also unable to examine risks in men.

## Conclusions

This study is the first to clearly quantify the relative and absolute risks of hip and knee joint replacement in middle-aged women in relation to body size in childhood, early adulthood and middle-age. Our results show that overall, body size in childhood and early adulthood has little effect on the cumulative risk of hip and knee joint replacement compared to BMI in middle-age. Applying the absolute risks from this study to UK population data [20] and survey data on BMI distribution in the population [21] suggests that about 40,000 of the 50,000 hip and knee replacements that occur annually in middle-aged UK women are attributable to overweight and obesity in middle-age. Even small reductions in the average weight of a population in middle-age should lead to appreciable reductions in hip and knee replacement rates and the associated health system costs.

## Abbreviations

BMI, body mass index; UK, United Kingdom; RR, relative risk

## Appendix

**Table 2:** Relative risk of hip and knee replacement by body mass index at around age 60 years cross-classified by birth weight, and body size at age 10 and 20 years

	Body mass index (kg/m^2^)	Relative risk of hip replacement (95 % CI ^a^)	Relative risk of knee replacement (95 % CI ^a^)
Size at age 10 years			
Small	<25	1.00 (0.95–1.05)	1.00 (0.94–1.06)
	25–29.9	1.37 (1.29–1.44)	2.65 (2.52–2.78)
	30+	1.80 (1.63–1.99)	6.49 (6.08–6.94)
Medium	<25	1.25 (1.20–1.30)	1.10 (1.05–1.16)
	25–29.9	1.60 (1.54–1.66)	2.61 (2.51–2.71)
	30+	2.06 (1.93–2.20)	6.18 (5.90–6.48)
Large	<25	1.61 (1.54–1.69)	1.43 (1.34–1.53)
	25–29.9	2.00 (1.93–2.07)	3.23 (3.12–3.35)
	30+	2.52 (2.42–2.62)	6.96 (6.76–7.18)
Size at age 10 years			
Thin	<25	1.00 (0.96–1.04)	1.00 (0.95–1.06)
	25–29.9	1.38 (1.32–1.44)	2.64 (2.54–2.74)
	30+	1.75 (1.64–1.87)	6.15 (5.89–6.42)
Average	<25	1.10 (1.07–1.14)	0.90 (0.86–0.95)
	25–29.9	1.52 (1.48–1.57)	2.26 (2.19–2.33)
	30+	2.03 (1.94–2.12)	5.35 (5.17–5.53)
Plump	<25	1.29 (1.20–1.39)	0.87 (0.78–0.97)
	25–29.9	1.67 (1.57–1.77)	2.08 (1.95–2.22)
	30+	2.40 (2.26–2.54)	5.25 (5.00–5.50)
Birth weight (kg)			
< 2.5	<25	1.00 (0.91–1.10)	1.00 (0.89–1.13)
	25–29.9	1.30 (1.19–1.41)	2.62 (2.42–2.84)
	30+	2.04 (1.84–2.26)	6.01 (5.57–6.49)
2.5–3.4	<25	0.96 (0.92–1.01)	0.93 (0.88–0.99)
	25–29.9	1.31 (1.26–1.37)	2.42 (2.32–2.52)
	30+	1.86 (1.75–1.97)	5.55 (5.31–5.80)
3.5+	<25	0.92 (0.87–0.98)	0.85 (0.78–0.92)
	25–29.9	1.31 (1.24–1.38)	2.28 (2.16–2.41)
	30+	1.79 (1.67–1.91)	5.56 (5.29–5.85)

^a^See methods regarding calculation of floating absolute risks
